# Focal sources of FGF-10 promote the buckling morphogenesis of the embryonic airway epithelium

**DOI:** 10.1242/bio.059436

**Published:** 2022-09-27

**Authors:** Kara E. Peak, Shelby R. Mohr-Allen, Jason P. Gleghorn, Victor D. Varner

**Affiliations:** ^1^ Department of Bioengineering, University of Texas at Dallas, Richardson, TX 75080, USA; ^2^ Department of Biomedical Engineering, University of Delaware, Newark, DE 19716, USA; ^3^ Department of Surgery, UT Southwestern Medical Center, Dallas, TX 75390, USA

**Keywords:** Lung development, Branching morphogenesis, Mechanobiology, Biomechanics, Chick embryo

## Abstract

During airway branching morphogenesis, focal regions of FGF-10 expression in the pulmonary mesenchyme are thought to provide a local guidance cue, which promotes chemotactically the directional outgrowth of the airway epithelium. Here, however, we show that an ectopic source of FGF-10 induces epithelial buckling morphogenesis and the formation of multiple new supernumerary buds. FGF-10-induced budding can be modulated by altered epithelial tension and luminal fluid pressure. Increased tension suppresses the formation of ectopic branches, while a collapse of the embryonic airway promotes more expansive buckling and additional FGF-10-induced supernumerary buds. Our results indicate that a focal source of FGF-10 can promote epithelial buckling and suggest that the overall branching pattern cannot be explained entirely by the templated expression of FGF-10. Both FGF-10-mediated cell behaviors and exogenous mechanical forces must be integrated to properly shape the bronchial tree.

## Teaser

**Summary:** In this paper, we show that ectopic sources of FGF-10 induce epithelial buckling and initiate the formation of new branches in cultured embryonic lungs.

## INTRODUCTION

Ramified networks constitute the basic architecture for many organs in the body, including the lung, kidney, and salivary gland (Lu and Werb, 2008). In the developing embryo, these structures arise via a process known as branching morphogenesis, in which an epithelial bud extends into a surrounding layer of mesenchyme and undergoes a series of branching events (Affolter et al., 2009; Varner and Nelson, 2014; Shamir and Ewald, 2014; Iber and Menshykau, 2013). Reciprocal signaling interactions between the mesenchyme and branching epithelium are essential to this process (Grobstein, 1953; Alescio and Cassini, 1962; Spooner and Wessells, 1970; Metzger and Krasnow, 1999; Hogan, 1999; Weaver et al., 2003) and are thought to give rise to a specific pattern of growth factor expression in the mesenchyme, which templates biochemically the overall branching pattern (Iber and Menshykau, 2013; Turing, 1952).

In the developing lung, the embryonic airway initiates as an out-pocketing of foregut endoderm, which then branches repeatedly to build the bronchial tree (Morrisey and Hogan, 2010). In the mammalian lung, this process involves distinct branching modes: lateral branches, which emerge along the length of a parent branch, and bifurcations, in which the tip of an epithelial branch splits to form two daughter branches (Metzger et al., 2008). Each of these modes depends crucially on signaling downstream of fibroblast growth factor 10 (FGF-10) (Metzger et al., 2008). Focal regions of FGF-10 expression in the pulmonary mesenchyme are thought to specify the locations of new epithelial branches (Park et al., 1998; Weaver et al., 2000), each individual spot of FGF-10 expression directing the formation of an individual bud (Iber and Menshykau, 2013).

Within this context, FGF-10 is believed to act as both a mitogen and chemoattractant. Isolated embryonic airway epithelial explants, for instance, grow toward FGF-10-loaded beads when cultured in three-dimensional (3D) gels of reconstituted basement membrane protein (Weaver et al., 2000), in a manner suggestive of chemotaxis. This idea is reinforced by the observation that similar FGF-10-loaded beads become enveloped by the branching airway epithelium in embryonic mouse lungs cultured *ex vivo* (Park et al., 1998). FGF-10, however, also regulates the proliferation of airway epithelial cells (Bellusci et al., 1997; Park et al., 1998; Weaver et al., 2000; Varner et al., 2015) and is thought to produce a local increase in proliferation that stimulates the formation of individual buds (Varner and Nelson, 2014). Such a mechanism is consistent with classic cut-and-paste embryological experiments, which have suggested that elevated proliferation is associated with the emergence of new branches (Goldin and Wessells, 1979; Goldin et al., 1984). But mechanical forces also influence airway branching (Nelson and Gleghorn, 2012; Varner and Nelson, 2014; Varner et al., 2015; Nelson et al., 2017; Tang et al., 2018; Morgan et al., 2018; Hogan, 2018; Goodwin and Nelson, 2020), and it remains unclear how these forces act in concert with the cellular behaviors downstream of FGF-10 to sculpt new embryonic airways.

Here, we focus on physical mechanisms of lateral branching morphogenesis and use as a model system the embryonic avian lung, which forms only lateral branches during early stages of development. We created an ectopic source of FGF-10 within the pulmonary mesenchyme of cultured embryonic lungs and used quantitative fluorescence microscopy, as well as experimental modulation of luminal fluid pressure, to assay the cellular and physical mechanisms induced along the airway epithelium by this focal source.

## RESULTS

### Individual focal sources of FGF-10 induce the formation of multiple ectopic branches

**Fig. 1.**
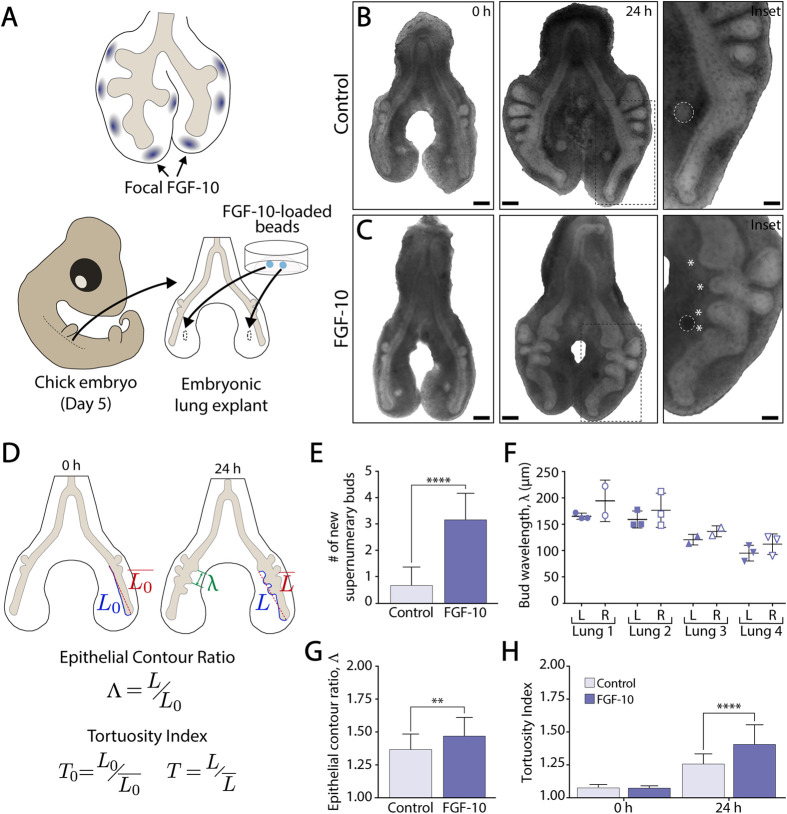
**Individual ectopic sources of FGF-10 elicit the formation of multiple supernumerary buds.** (A) Schematic representation of the spatial pattern of FGF-10 expression in the embryonic mouse lung (top), as well as the implantation of ectopic sources of FGF-10 within the pulmonary mesenchyme of embryonic avian lungs (bottom). (B,C) Bright-field images of representative embryonic lung explants cultured *ex vivo* with either (B) PBS- or (C) FGF-10-loaded beads. White dashed lines indicate the location of the bead. White asterisks indicate supernumerary buds. Scale bars: 200 µm; inset scale bars: 100 µm. (D) Schematic representation of metrics used to quantify epithelial morphology. (E-H) Quantification of (E) the number of supernumerary buds, (F) the bud wavelength *λ*, (G) the epithelial contour ratio Λ=*L*/*L*_0_, and (H) the tortuosity index 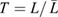 for explants cultured with either PBS- or FGF-10 loaded beads, where *L* is the measured length of the ventral epithelium at each time-point, 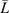 is the length of the straight line connecting the endpoints of *L*, and *L*_0_ is the length of the ventral epithelium at *t*=0 h, which is used as a reference length. A Student's unpaired *t*-test (E,G) or a one-way ANOVA (H), followed by a Tukey *post-hoc* test, were used to make statistical comparisons. (PBS: *n*=29, FGF-10: *n*=37; *P<0.05, **P<0.01, ***P<0.001, ****P<0.0001; error bars represent s.d.).

We implanted agarose beads containing recombinant FGF-10 into the pulmonary mesenchyme of embryonic lungs explanted from Hamburger and Hamilton (HH) stage 26 chicken embryos (Hamburger and Hamilton, 1951). Individual beads had a diameter on the scale of new epithelial buds and were placed adjacent to the ventral airway epithelium, a region of the primary bronchus that remains unbranched during early stages of lung development (Gleghorn et al., 2012; Locy and Larsell, 1916). Each lung was then cultured *ex vivo* for 24 h to focus on bud initiation (Fig. 1A; Fig. S1). To our surprise, individual FGF-10-loaded beads induced the formation of multiple supernumerary buds (indicated by white asterisks), while control beads soaked in phosphate-buffered saline (PBS) failed to elicit any ectopic buds (Fig. 1B,C). Normal buds, located along the dorsal aspect of the primary bronchus, appeared unaffected by the ectopic sources of FGF-10 (Fig. S2). In contrast, explants cultured in the presence of ubiquitous FGF-10 formed ectopic buds distributed along the entire bronchial tree (Fig. S3). Within explants containing an FGF-10-loaded bead, quantitative morphological measurements revealed a significant increase in both the number of supernumerary buds, as well as the length and tortuosity of the ventral epithelium (Fig. 1D-H). Notably, the ectopic buds formed with a characteristic wavelength, which varied in magnitude from lung to lung but was remarkably conserved within each explant (Fig. 1F). Lungs cultured in the presence of either the FGF receptor (FGFR) inhibitor SU5402 (Fig. S4) or the MAP kinase (MAPK) inhibitor U0126 (Fig. S5) confirmed that the formation of supernumerary buds depended on signaling downstream of FGF-10. Both inhibitors suppressed ectopic budding (Fig. S4A-D, Fig. S5A-D) and produced a shorter, less tortuous ventral epithelium, similar to that observed in controls (Fig. S4E,F, Fig. S5E,F).

**Fig. 2.**
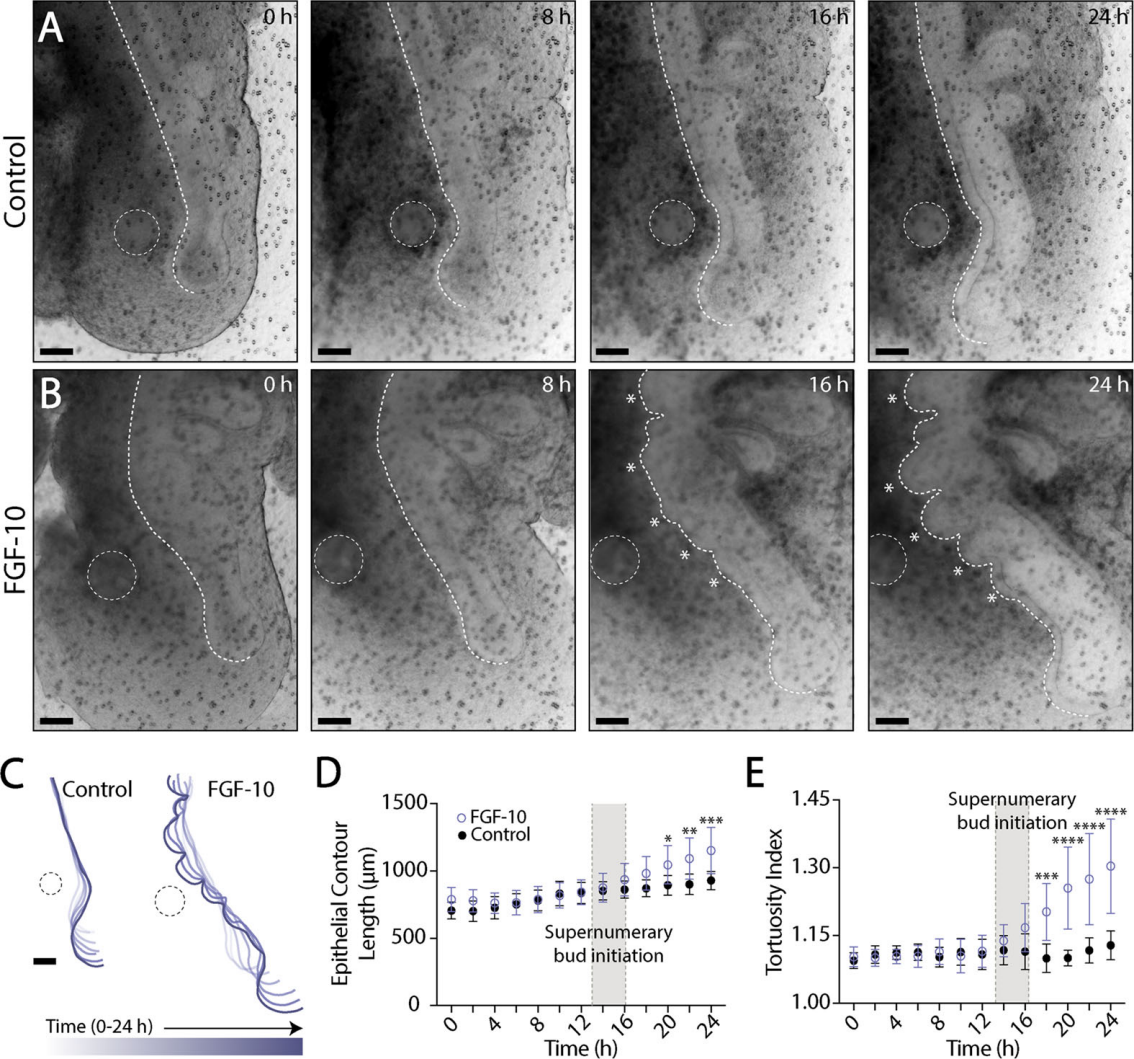
**FGF-10-induced supernumerary branches form simultaneously.** (A,B) Bright-field images of a representative embryonic lung explant cultured *ex vivo* with either a (A) PBS- or (B) FGF-10-loaded bead over 24 h of time-lapse culture. White dashed lines indicate the location of the bead, as well as the traced contour of the ventral epithelium. White asterisks indicate supernumerary buds. Scale bars: 100 µm. (C) Time-varying contours of the ventral epithelium over 24 h of time-lapse culture. Scale bar: 100 µm. (D,E) Quantification of (D) epithelial contour length and (E) tortuosity index (E) during time-lapse culture. Shaded gray regions indicate the initiation of supernumerary buds. A two-way ANOVA, followed by a Tukey *post-hoc* test, was used to determine significance among groups. (Control: *n*=7; FGF-10: *n*=12; *P<0.05, **P<0.01, ***P<0.001, ****P<0.0001; error bars represent s.d.).

During normal airway branching, lateral buds emerge sequentially in a proximo-distal fashion along the length of the primary bronchus (Metzger et al., 2008; Gleghorn et al., 2012; Locy and Larsell, 1916). To investigate the dynamics of supernumerary budding morphogenesis, we cultured explants containing FGF-10-loaded beads in a stage-top incubator and captured time-lapse images every 30 min for 24 h of culture. Somewhat unexpectedly, the supernumerary buds formed simultaneously after approximately 15 h of culture and then grew outward into the surrounding mesenchyme. (Fig. 2A-C; Movie 1). Once the buds had formed, their relative spacing did not change (Fig. 2C). The length and tortuosity of the ventral epithelium, however, increased monotonically with time, differing significantly from control explants by approximately 20 h of culture (Fig. 2D,E).

### Elevated levels of proliferation required for supernumerary bud formation

**Fig. 3.**
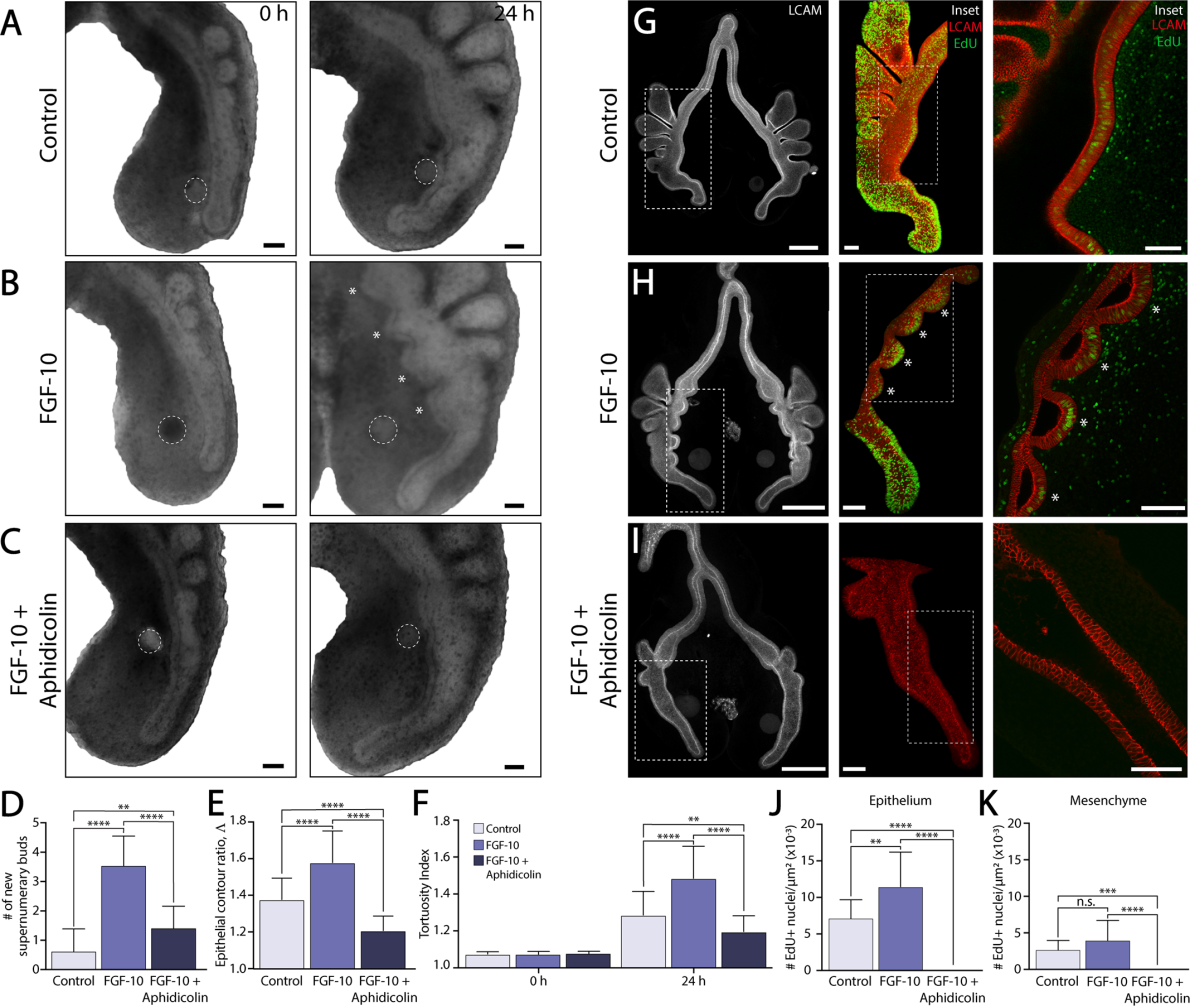
**Supernumerary bud formation depends crucially on elevated epithelial proliferation.** (A-C) Bright-field images of representative embryonic lung explants cultured *ex vivo* with either a (A) PBS- or (B,C) FGF-10-loaded bead. Some explants were also treated with (C) the cell cycle inhibitor, aphidicolin. White dashed lines indicate the location of the bead. White asterisks indicate supernumerary buds. Scale bars: 100 µm. (D-F) Quantification of (D) the number of new supernumerary buds, (E) epithelial contour ratio, and (F) tortuosity index for explants cultured with either a PBS- or FGF-10-loaded bead in the presence or absence of aphidicolin. A (D,E) one-way or (F) two-way ANOVA, followed by a Tukey *post-hoc* test, was used to make statistical comparisons. (PBS: *n*=32, FGF-10: *n*=36, FGF-10+Aphidicolin: *n*=29; *P<0.05, **P<0.01, ***P<0.001, ****P<0.0001; error bars represent s.d.). (G-I) Confocal images of EdU incorporation and LCAM/E-cadherin immunofluorescence in representative whole-mount lung explants. Insets show 3D reconstructions of EdU-positive nuclei within the embryonic airway epithelium. White asterisks denote elevated proliferation in supernumerary buds. Scale bars: 200 µm; inset scale bars: 50 µm. (J,K) Quantification of EdU incorporation within (J) the ventral epithelium and (K) adjacent pulmonary mesenchyme. A one-way ANOVA, followed by a Tukey *post-hoc* test, was used to determine significance between groups (PBS: *n*=13, FGF-10: *n*=13, FGF-10+Aphidicolin: *n*=12; **P<0.01, ***P<0.001, ****P<0.0001, error bars represent s.d.).

The simultaneity of supernumerary budding, along with the observed characteristic wavelength, was reminiscent of the morphogenesis of isolated embryonic airway epithelial explants, which, when cultured in 3D gels of reconstituted basement membrane protein, branch simultaneously in a proliferation-dependent manner (Varner et al., 2015). To determine the role of proliferation during supernumerary bud formation, we cultured explants containing FGF-10-loaded beads in the presence or absence of the cell-cycle inhibitor aphidicolin. Treatment with aphidicolin completely blocked the formation of FGF-10-induced supernumerary buds (Fig. 3A-C). In these explants, the ventral epithelium exhibited reduced elongation and tortuosity, as compared to untreated FGF-10-containing controls, and was morphologically similar to the ventral epithelium in explants containing only PBS-loaded beads (Fig. 3D-F).

To quantify the patterns of proliferation induced by individual FGF-10-loaded beads, we assayed patterns of 5-ethynyl-2′-deoxyuridine (EdU) incorporation in explants that were pulsed with EdU after 24 h of culture. Samples were then fixed, co-stained for E-cadherin immunofluorescence, optically cleared, and imaged as whole mounts (Fig. 3G-I). In each confocal image, E-cadherin staining was used to segment the population of EdU-positive nuclei located within the airway epithelium. Three-dimensional reconstructions of segmented confocal images revealed distinct patterns of proliferation along the embryonic airway epithelium (Fig. 3G-I; Movies 2-4). In explants cultured with only PBS-soaked beads, high levels of EdU incorporation were observed within normal buds and in the distal tip of the primary bronchus, but no consistent distribution of EdU-positive nuclei was visible along the ventral epithelium (Fig. 3G). Each FGF-10-loaded bead, however, elicited elevated levels of EdU incorporation within each of the supernumerary buds, with the highest levels of proliferation localized to the bud tips (Fig. 3H). In explants treated with aphidicolin, no EdU-positive nuclei were observed in either the epithelium or mesenchyme, confirming that proliferation had been inhibited (Fig. 3I-K). Quantitative image analysis revealed a significant increase in EdU incorporation within the ventral epithelium of explants containing an FGF-10-loaded bead, as compared to either untreated controls or explants cultured in the presence of aphidicolin; no concomitant increase in proliferation was observed within the pulmonary mesenchyme (Fig. 3J,K). In addition, just prior to the formation of supernumerary buds (at approximately 12 h of culture), no distinct distribution of proliferating cells could be detected along the ventral epithelium in FGF-10-treated explants, suggesting that a repeating pattern of localized proliferation does not precede the emergence of ectopic buds (Fig. S6A,B). Rather, each FGF-10-loaded bead induced a relatively uniform pattern of proliferation along the ventral epithelium, which then promoted the formation of multiple supernumerary branches.

### Apical constriction is not associated with the formation of supernumerary buds

**Fig. 4.**
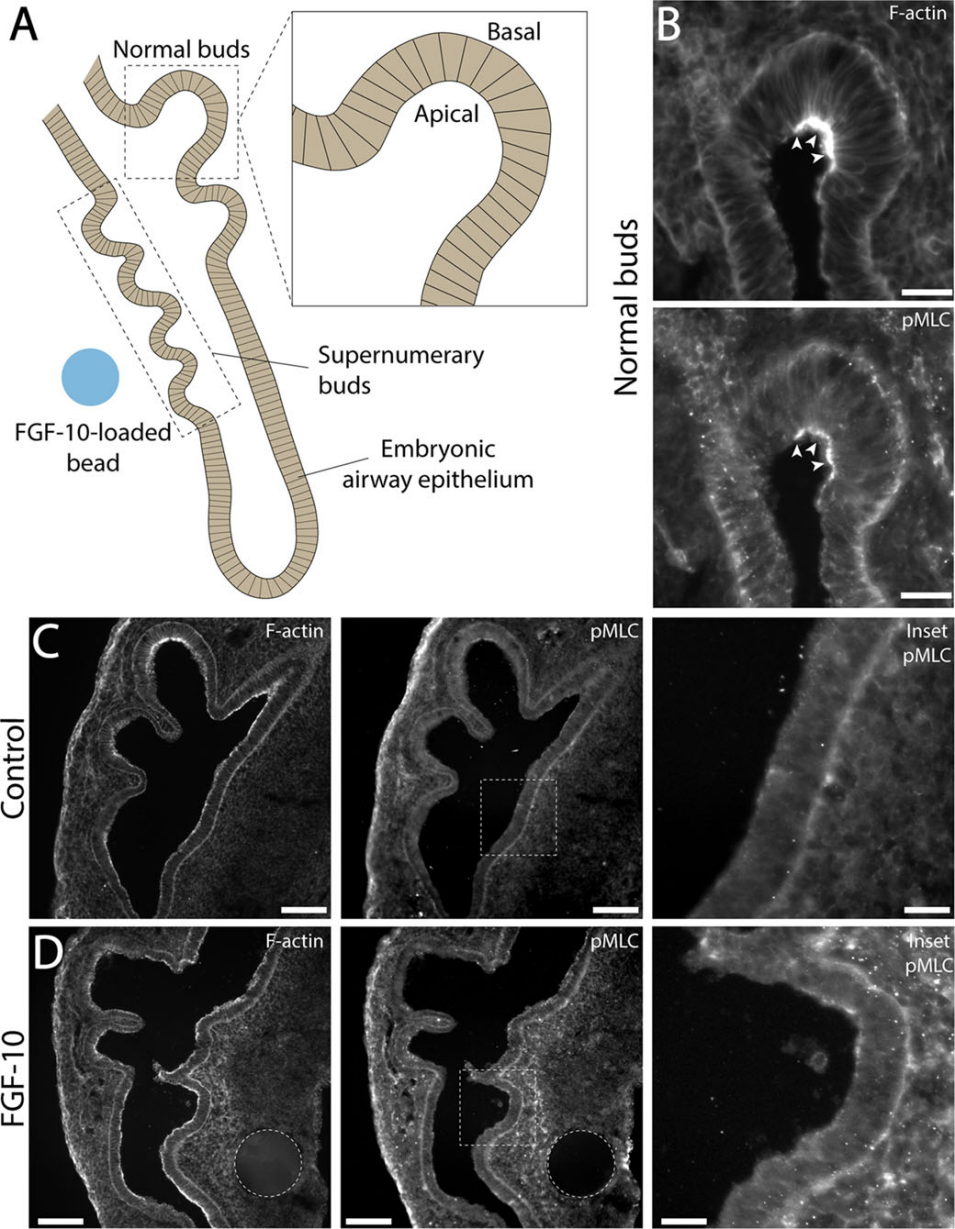
**Apical constriction is not associated with the formation of FGF-10-induced supernumerary buds.** (A) Schematic depiction of airway epithelial cells within both normal and supernumerary buds. (B) Wide-field fluorescence images of F-actin and pMLC staining within normal epithelial buds. White arrowheads indicate elevated fluorescence intensity along the apical surface. Scale bars: 25 µm. (C,D) Wide-field fluorescence images of F-actin and pMLC staining in representative embryonic lungs cultured with either (C) PBS- or (D) FGF-10-loaded beads. Insets depict staining within the ventral epithelium at higher magnification. Scale bars: 100 µm; inset scale bars: 25 µm.

Actomyosin contractility contributes to the initiation of normal buds during mammalian and avian lung development (Kim et al., 2013; Kadzik et al., 2014). F-actin and non-muscle myosin-II become localized apically within airway epithelial cells to produce changes in cell shape that cause the epithelium to fold and form new lateral branches (Kim et al., 2013). To determine if apical constriction is associated with the formation of FGF-10-induced supernumerary buds (Fig. 4A), we stained explants cultured with FGF-10-loaded beads for both F-actin and phosphorylated myosin light chain (pMLC) immunofluorescence. In normal epithelial buds, we observed apical co-localization of both F-actin and pMLC, consistent with previous work (Kim et al., 2013) (Fig. 4B). No discernible increases in the intensity of either F-actin or pMLC, however, were observed along the apical membrane of cells within FGF-10-induced supernumerary buds (Fig. 4C,D). In these explants, the patterns of F-actin and pMLC immunofluorescence were indistinguishable from controls (Fig. 4C,D), suggesting that the apical constriction of airway epithelial cells is not involved in the formation of supernumerary branches.

### Altered luminal fluid pressure modulates FGF-10-induced supernumerary budding

**Fig. 5.**
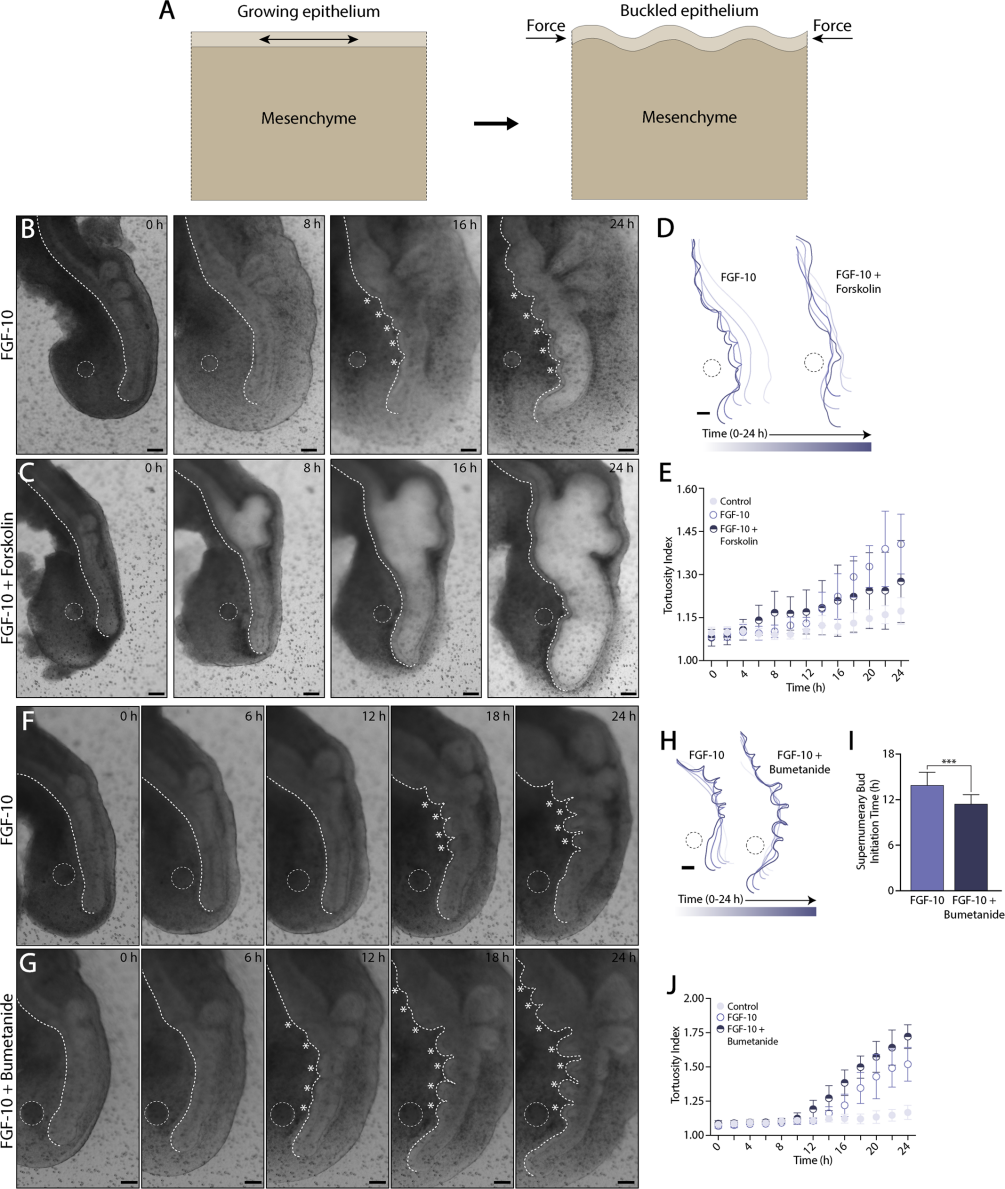
**Altered epithelial tension modulates supernumerary bud formation.** (A) Schematic of buckling morphogenesis. Epithelial growth, constrained by the surrounding mesenchyme, can cause the epithelium to buckle and form new buds. (B,C) Bright-field time-lapse images of representative lung explants cultured *ex vivo* for 24 h with an FGF-10-loaded bead. Some explants (C) were treated with forskolin, an agonist of epithelial fluid secretion. Scale bars: 100 µm. White dashed lines indicate the location of the bead, as well as the traced contour of the ventral epithelium. White asterisks denote supernumerary buds. (D) Time-varying contours of the ventral epithelium over 24 h of time-lapse culture in either the presence or absence of forskolin. Scale bar: 100 µm. (E) Quantification of the tortuosity index during time-lapse culture. (PBS: *n*=10; FGF-10: *n*=11; FGF-10+10 µM forskolin: *n*=13; error bars represent s.d.) (F,G) Bright-field time-lapse images of representative lung explants cultured *ex vivo* for 24 h with an FGF-10-loaded bead. Some explants (G) were treated with bumetanide, an inhibitor of epithelial fluid secretion. Scale bars: 100 µm. As above, white dashed lines indicate the location of the bead, as well as the traced contour of the ventral epithelium. White asterisks denote supernumerary buds. (H) Time-varying contours of the ventral epithelium over 24 h of time-lapse culture in either the presence or absence of bumetanide. Scale bar: 100 µm. (I) Quantification of the time at which supernumerary buds initiate. A Students’ *t*-test was used to determine significance between groups. (FGF-10: *n*=43; FGF-10+200 µM bumetanide: *n*=37; *P<0.05, **P<0.01, ***P<0.001, ****P<0.0001; error bars represent s.d.). (J) Quantification of the time-varying tortuosity index within explants cultured with either PBS- or FGF-10-loaded beads in the presence or absence of bumetanide. (PBS: *n*=10; FGF-10: *n*=10; FGF-10+200 µM bumetanide: *n*=12; error bars represent s.d.).

Thus far, our data indicate that an ectopic source of FGF-10 can promote the simultaneous formation of multiple supernumerary buds, which arise via changes in proliferation and exhibit a characteristic wavelength. These observations are consistent with the previously reported buckling morphogenesis of embryonic airway epithelial explants cultured in gels of reconstituted basement membrane protein (Varner et al., 2015). Within intact embryonic lungs, however, FGF-10-induced epithelial growth is constrained mechanically by the surrounding pulmonary mesenchyme (Fig. 5A). To test this buckling idea more directly, we used pharmacological modulators of epithelial fluid secretion to alter luminal fluid pressure in explants cultured with an FGF-10-loaded bead (Fig. 5B,C) (Narayanan et al., 2020). Increases in tension would be expected to reduce epithelial buckling, and thus supernumerary bud formation, while decreases in tension would be expected to do the opposite. Treatment with forskolin, an agonist of epithelial fluid secretion (Ballard and Spadafora, 2007; Ballard et al., 2006), caused the embryonic airways to stretch and dilate dramatically (Fig. 5B,C). (Explants cultured with PBS-loaded beads underwent a similar forskolin-mediated dilation.) To qualitatively assess how forskolin affects luminal pressure, we used a microneedle to puncture the airway, which caused a complete collapse of the primary bronchus, a result that suggests treatment with forskolin increases both luminal pressure and epithelial tension (Fig. S7). This forskolin-mediated dilation disrupted FGF-10-induced supernumerary budding (Fig. 5D; Fig. S8A-D, Movie 5), and quantitative morphological measurements of the ventral airway epithelium indicated an increase in length, consistent with epithelial stretching (Fig. S8E), as well as a decrease in tortuosity (Fig. 5E; Fig. S8F). Interestingly, treatment with forskolin also suppressed the formation of normal buds located along the dorsal aspect of the primary bronchus.

In other experiments, explants containing an FGF-10-loaded bead were cultured in the presence of bumetanide, an antagonist of fluid secretion (McCray and Welsh, 1991; Souza et al., 1995). Treatment with bumetanide caused the embryonic airways to collapse (Fig. S9A,B) and, in the presence of an FGF-10-loaded bead, promoted an increase in supernumerary branching, as compared to untreated controls (Fig. S10A-D). Time-lapse culture revealed that FGF-10-induced supernumerary buds formed earlier in the presence of bumetanide and were more expansively distributed along the length of the primary bronchus, observations consistent with an epithelial buckling mechanism (Fig. 5F-J; Movie 6). This increase in supernumerary budding was associated with a shorter branch wavelength (Fig. S10E), as well as a longer and more tortuous ventral epithelium when compared to controls (Fig. 5J; Fig. S10F,G). Taken together, these experiments suggest that a focal source of FGF-10 can promote the buckling morphogenesis of the airway epithelium, resulting in the formation of multiple nascent buds (Fig. S11A), a process that can be modulated by changes in luminal fluid pressure (Fig. S11B).

## DISCUSSION

Numerous signaling pathways, including those downstream of Sonic hedgehog (Shh), FGF, and Wnt, among others, regulate the formation of the bronchial tree during embryonic lung development (Morrisey and Hogan, 2010; Cardoso and Lü, 2006; Warburton et al., 2000; Jones et al., 2021). It is thought that these pathways give rise to a distinct spatial pattern of FGF-10 expression in the pulmonary mesenchyme, which then serves as a biochemical template for the overall branching pattern, with individual spots of FGF-10 expression eliciting the formation of individual branches (Metzger and Krasnow, 1999; Morrisey and Hogan, 2010; Park et al., 1998; Bellusci et al., 1997; Jones et al., 2021). Several recent studies, however, have shown that mechanical forces can also influence airway branching (Varner and Nelson, 2014; Varner et al., 2015; Goodwin and Nelson, 2020; Nelson and Gleghorn, 2012; Warburton et al., 2010; Hogan, 2018; Tang et al., 2018). Microfluidic control of luminal fluid pressure, for instance, alters rates of branching morphogenesis in cultured embryonic mouse lungs (Nelson et al., 2017), perhaps by modulating levels of FGF-10 expression (Stanton et al., 2021) or retinoic acid signaling (Jaslove et al., 2022). But it is still unclear how the cellular behaviors downstream of FGF-10 act in concert with exogeneous mechanical forces to sculpt new epithelial branches.

Here, we used growth-factor-loaded beads to create ectopic sources of FGF-10 within the pulmonary mesenchyme and investigated the response induced within the airway epithelium by these individual focal sources. To our surprise, each FGF-10-loaded bead elicited the formation of multiple supernumerary buds. These buds formed simultaneously with a characteristic wavelength and depended crucially on elevated proliferation in the airway epithelium, observations suggestive of an epithelial buckling mechanism, as has been observed during the morphogenesis of isolated airway epithelial explants in 3D gels of reconstituted basement membrane protein (Varner et al., 2015). To test this buckling idea more directly, we used pharmacological modulators of epithelial fluid secretion to alter luminal fluid pressure (and thereby epithelial tension) in explants cultured *ex vivo*. Supernumerary budding was disrupted if the embryonic airways were dilated using an agonist of fluid secretion and enhanced if the airways were collapsed using an inhibitor of secretion. Since increased tension would be expected to suppress the onset of buckling, while a collapse of the primary bronchus would be expected to promote it, these results were consistent with an FGF-10-induced buckling phenotype. Taken together, our data highlight the importance of exogenous mechanical forces during airway branching and suggest that a focal source of FGF-10 can promote the buckling morphogenesis of the embryonic airway epithelium.

Previous studies have primarily ascribed a chemotactic role to FGF-10 during lung development (Park et al., 1998; Weaver et al., 2000). Focal regions of FGF-10 are thought to provide a local guidance cue, which stimulates the directional outgrowth of a specific population of airway epithelial cells, in a manner akin to the role of the FGF and FGF receptor homologues, Branchless and Breathless, during *Drosophila* tracheal morphogenesis (Sutherland et al., 1996; Metzger and Krasnow, 1999). Since FGF-10 is also a potent mitogen (Igarashi et al., 1998), numerous studies have suggested that FGF-10 also induces localized proliferation to initiate the formation of new buds (Weaver et al., 2000; Schnatwinkel and Niswander, 2013). Clear experimental evidence for a pre-pattern of proliferative cells prior to the formation of new branches, however, has been lacking (Varner and Nelson, 2014; Nogawa et al., 1998), and the idea that proliferation elicited by FGF-10 might promote an epithelial buckling event has not been explored previously within intact embryonic lungs.

During normal lateral branching morphogenesis, new buds emerge sequentially along the length of the primary bronchus (Metzger et al., 2008; Gleghorn et al., 2012), a time-course which suggests that any FGF-10-induced buckling deformations are somehow constrained spatiotemporally. In the developing mouse lung, a layer of smooth muscle cells differentiates adjacent to the branching airway epithelium (Sparrow and Lamb, 2003; Tollet et al., 2001), and spatiotemporal patterns of smooth muscle wrapping have been shown to influence the morphogenesis of both lateral branches (Goodwin et al., 2019) and bifurcations (Kim et al., 2015). The presence of smooth muscle is thought to locally inhibit bud formation, causing new branches to emerge in locations where smooth muscle has not yet differentiated (Kim et al., 2015; Goodwin et al., 2019). It may be that this stiff layer of smooth muscle spatially constrains any FGF-10-mediated epithelial buckling. Indeed, consistent with this idea, disrupted smooth muscle differentiation elicits a buckling-like phenotype in cultured embryonic mouse lungs and interferes with the sequential emergence of new lateral branches (Goodwin et al., 2019). In the developing avian lung, smooth muscle is not yet present at the stages investigated here (Spurlin et al., 2019; Palmer and Nelson, 2020), which may explain, in part, why individual ectopic sources of FGF-10 elicit epithelial buckling and the formation of multiple supernumerary buds. It would be interesting to explore directly whether a stiffening of the pulmonary mesenchyme can locally suppress FGF-10-induced budding.

It also remains a possibility that cell-ECM interactions influence FGF-10-mediated buckling. Recent experiments in the embryonic salivary gland, for instance, have shown that strong cell-ECM (and low cell–cell) adhesions can promote cleft formation and budding morphogenesis (Wang et al., 2021). In addition, previous work in the developing mammary gland has indicated that local accumulations of ECM can influence patterns of epithelial branching (Nerger et al., 2021). Still, it is unclear if similar mechanisms contribute to the morphogenesis of FGF-10-induced supernumerary buds in the embryonic lung.

In addition, although our data suggest that an increase in fluid pressure can suppress FGF-10-mediated budding, prior investigations, using cultured embryonic mouse lungs, have found that elevated luminal pressure increases rates of branching morphogenesis (Unbekandt et al., 2008; Nelson et al., 2017). The reason for these divergent results is not necessarily clear. Although forskolin is a well-established agonist of fluid secretion in the developing lung (Ballard et al., 2006), it is a possibility that cAMP activation has other cell-specific effects that contribute to the observed phenotype. There are also, of course, important species-specific differences between avian and murine lungs, and it could be that different branching modes (e.g. lateral branches, bifurcations) are affected differently by changes in luminal pressure, or that transmural pressure somehow regulates smooth muscle differentiation, which then constrains any FGF-10-mediated buckling. In support of this second possibility, recent work has shown that luminal fluid pressure can impact smooth muscle wrapping in embryonic mouse lungs via changes in retinoic acid signaling (Jaslove et al., 2022). Future experiments, which use more direct means to modulate fluid pressure, will be needed to investigate the possibility of species-specific differences in the role of transmural pressure during lung development.

In either case, taken together, our data suggest that the airway branching pattern cannot be explained completely by the templated expression of FGF-10. Previous work has suggested that FGF-10 is expressed in a focal pattern within the pulmonary mesenchyme of both embryonic mouse and chicken lungs (Metzger and Krasnow, 1999; Sakiyama et al., 2003; Moura et al., 2011; Jones et al., 2021). Our data, however, indicate that individual sources of FGF-10 can elicit the formation of multiple supernumerary buds, which arise via bucking morphogenesis. The onset of FGF-10-induced buckling can be modulated by changes in fluid secretion, highlighting the role of mechanical forces during this process. The physical mechanisms that sculpt the bronchial tree are complex and involve both FGF-10-mediated cell behaviors, as well as exogenous mechanical forces. Endogenous cellular processes, such as proliferation, must be kept in balance with biophysical factors, such as luminal fluid pressure or mechanical inputs from the pulmonary mesenchyme, to build reproducibly a stereotyped bronchial tree.

## MATERIALS AND METHODS

### Preparation of growth factor-loaded beads

Small agarose beads approximately 75-150 µm in diameter (Affi-Gel Blue Media, Bio Rad) were rinsed overnight in PBS at 4°C. The beads were then placed in small droplets of either PBS or 100 µg/ml recombinant human FGF-10 (R&D Systems, 345-FG) in PBS and incubated for 2-3 h at room temperature.

### *Ex vivo* culture of embryonic lungs

Fertilized White Leghorn chicken eggs were incubated in a humidified forced-draft incubator at 37°C until HH stage 26 (Hamburger and Hamilton, 1951). Embryonic lungs were then dissected under a stereomicroscope (Olympus, SZX7; Waltham, MA, USA) using fine forceps in PBS supplemented with antibiotics (50 U/ml penicillin/streptomycin, Invitrogen; Carlsbad, CA, USA). A small incision was made in the pulmonary mesenchyme of each lobe, along the ventral aspect of the primary bronchus. A single growth-factor-loaded (or control) bead was carefully placed in each incision using fine forceps. Embryonic lung explants were then cultured at the fluid-air interface for 24 h at 37°C on Nucleopore membranes (diameter: 25 mm; pore size: 8 µm) in DMEM/F12 medium (without HEPES) that had been supplemented with 5% fetal bovine serum (FBS, heat inactivated) and antibiotics (50 U/ml penicillin/streptomycin) (Gleghorn et al., 2012). In some experiments, the culture medium was also supplemented with either 1 µM aphidicolin (Sigma-Aldrich; St. Louis, MO, USA), 5 µM SU5402 (Sigma-Aldrich; St. Louis, MO, USA), or 50 µM U0126 (Promega; Madison, WI, USA) to inhibit either cell proliferation, FGF receptor, or MAP kinase, respectively. Other explants were treated with either 10 µM forskolin (Tocris; Bristol, UK) or 200 µM bumetanide (Sigma-Aldrich) to either activate or inhibit epithelial fluid secretion, respectively. Bright-field images of cultured lung explants were captured at 0 and 24 h using a Zeiss AxioVert microscope equipped with a 5×, NA 0.15, LD A-Plan objective (Zeiss).

### Time-lapse microscopy

Some explants were cultured in a humidified stage incubator, situated atop a Zeiss AxioObserver7 microscope equipped with a motorized stage. Bright-field images were captured at 30 min intervals for 24 h of culture using a 5×, NA 0.16, Plan-Apochromat objective (Zeiss).

### Immunofluorescence staining and imaging

Cultured explants were fixed in 4% paraformaldehyde in PBS for 15 min at room temperature, then permeabilized and blocked in a solution of 10% goat serum and 0.5% Triton X-100 in PBS (PBST) for 1 h at room temperature. The Click-iT EdU Imaging Kit (Invitrogen) was used to detect cells in S-phase. (All explants were pulsed with EdU for 20 min prior to fixation.) Afterward, samples were incubated with primary antibody overnight on a shaker at 4°C. The following primary antibodies were used: anti-L-CAM/E-cadherin (1:100 dilution, 7D6; Developmental Studies Hybridoma Bank) and anti-phospho-myosin light chain (pMLC) 2 (1:50 dilution, Ser19, Cell Signaling Technology; Danvers, MA, USA). After extensive washing, samples were incubated with Alexa Fluor-conjugated secondary antibody and/or Alexa Fluor 594 phalloidin (1:200 dilution, Invitrogen). Stained explants were then dehydrated in a graded methanol series and optically cleared with Murray's clear. Samples were then imaged as whole mounts using a Zeiss LSM 800 laser scanning confocal microscope with a 20×, NA 0.8, Plan-Apochromat objective (Zeiss). Other samples were fixed as described above, dehydrated overnight in a 30% solution of sucrose in PBS, and then frozen in optimal cutting temperature (OCT) compound. Frozen sections, 20 µm in thickness, were obtained using a cryostat (Leica, CM1860). Sections were stained as described above and imaged with a Zeiss AxioVert microscope using a 20×, NA 0.35, LD A-Plan objective (Zeiss).

### Quantitative image analysis

Bright-field images of cultured explants were used to quantify changes in epithelial geometry. Epithelial contours were traced manually in ImageJ at each time-point. Relative changes in epithelial length were quantified using the contour ratio Λ=*L*/*L*_0_, where *L* is the measured length of the ventral epithelium at each time-point and *L*_0_ is the length at t=0 h, which was used as a reference length. The tortuosity (or waviness) of the ventral epithelium was measured using the tortuosity index *T*, defined at each time-point as the ratio of the epithelial contour length *L* and the length of a straight line 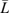 connecting the end-points of *L*, such that 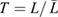. Three-dimensional reconstructions of confocal stacks were created in Imaris (Bitplane), and cell proliferation was quantified using the Cell Counter plugin in ImageJ.

### Statistical analysis

Data represent means±s.d. for at least three independent experiments. Statistical comparisons were made using either a Student's *t*-test, or a one- or two-way ANOVA, followed by a Tukey *post-hoc* test, with *P*-values as specified in the figure legends.

## Supplementary Material

10.1242/biolopen.059436_sup1 Supplementary information
