# Supplementary material for: Focal sources of FGF-10 promote the buckling morphogenesis of the embryonic airway epithelium
Source: Biol Open. 2022 Sep 27;11(9):bio059436. doi: 10.1242/bio.059436 (PMC9536751; doi:10.1242/bio.059436)
Supplement: Supplementary information [file biolopen-11-059436-s1.pdf]

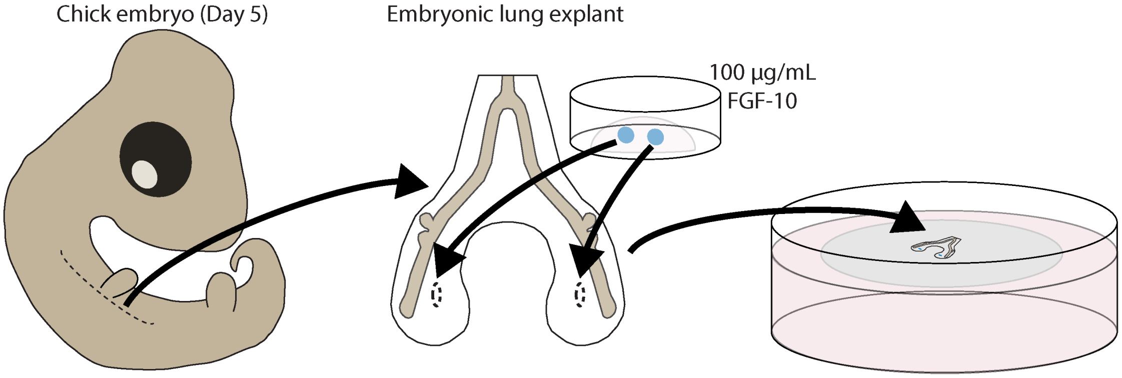

Fig. S1. Implanting ectopic sources of FGF-10 within cultured embryonic lungs. Embryonic lung explants are dissected from chicken embryos at embryonic day 5. Agarose beads soaked in either PBS or 100 µg/mL FGF-10 are then placed into the pulmonary mesenchyme along regions of the ventral epithelium that normally remain unbranched. Explants are then cultured ex vivo at the fluid-air interface.

Biology Open • Supplementary information

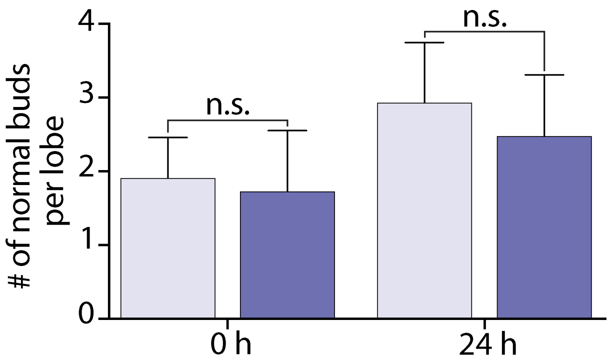

Fig. S2. Normal bud formation unaffected by focal sources of FGF-10. Quantification of the number of normal buds per lobe at 0 and 24 hr in lungs cultured ex vivo with either PBS- or FGF-10-loaded beads. A two-way ANOVA was used to determine significance. (PBS: $n = 11$, FGF-10: $n = 14$, * $p < 0.05$, ** $p < 0.01$, *** $p < 0.001$, **** $p < 0.0001$; error bars represent s.d.).

Biology Open • Supplementary information

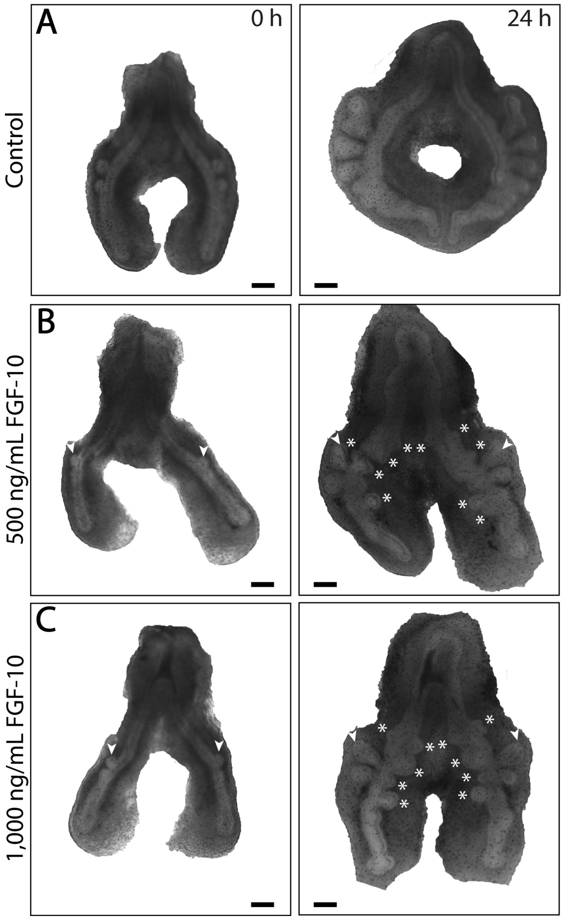

Fig. S3. Ubiquitous treatment with exogenous FGF-10 promotes broad ectopic branch-ing. (A-C) Bright-field images of representative lung explants cultured ex vivo in culture medium supplemented with exogenous FGF-10. White asterisks indicate supernumerary buds. White ar-rowheads indicate the location of the first normal bud. Note that, in the presence of ubiquitous FGF-10, several supernumerary buds form proximal to this first normal bud. Scale bars, 200 µm. (Control: n = 8, 500 ng/mL FGF-10: n = 8, 1,000 ng/ml FGF-10: n = 8).

Biology Open • Supplementary information

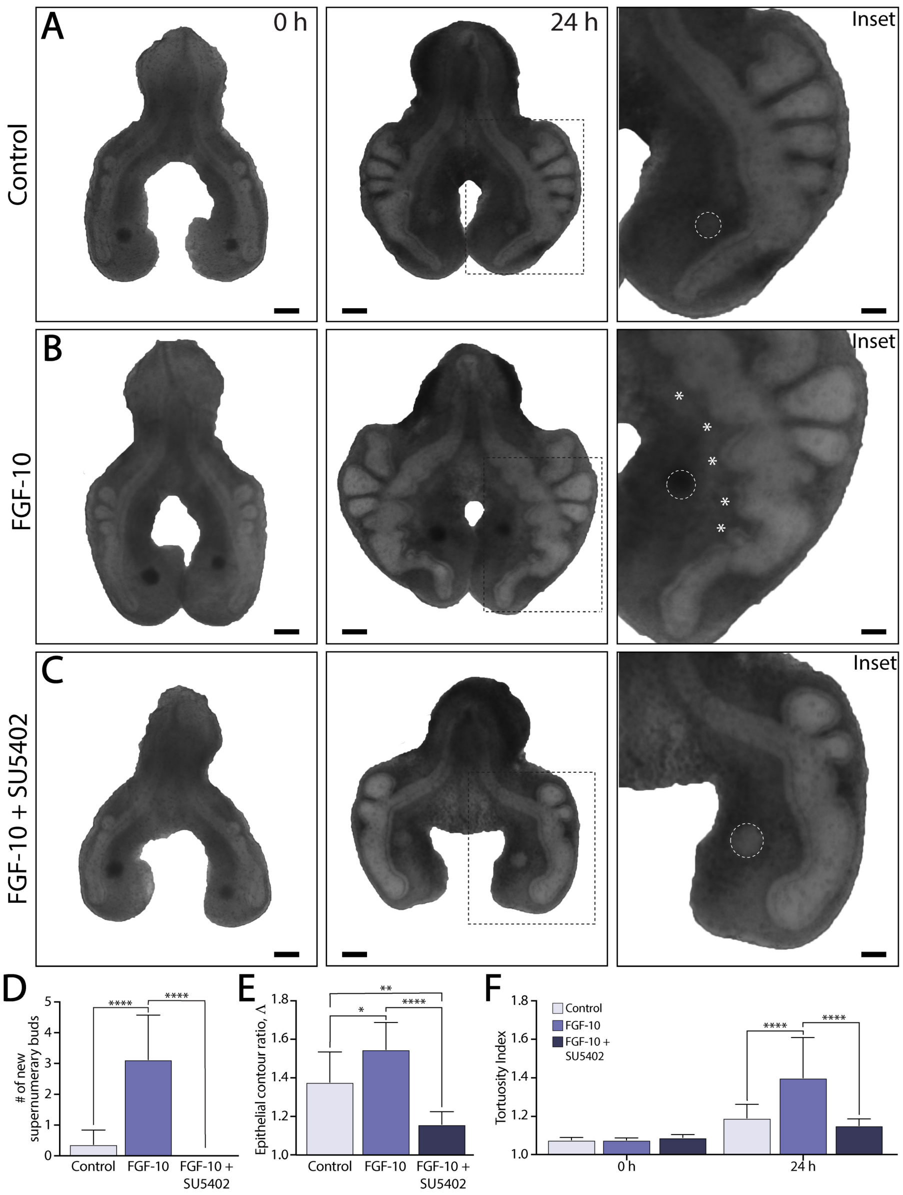

Fig. S4. Inhibiting FGFR blocks the formation of supernumerary buds. (A-C) Bright-field images of representative lung explants cultured ex vivo with either (A) PBS- or (B-C) FGF-10-loaded beads. Some explants (C) were treated with the FGF-receptor inhibitor SU5402. White dashed lines indicate the locations of growth-factor-loaded beads. White asterisks indicate supernumerary buds. Scale bars, 200 µm. (Inset scale bars, 100 µm.) (D-F) Quantification of (D) the number of supernumerary buds, (E) epithelial contour ratio, and (F) tortuosity index. A one-way (D-E) or two-way ANOVA (F), followed by a Tukey post-hoc test, was used to make statistical comparisons.(PBS: n = 10, FGF-10: n = 12; FGF-10 + SU5402: n = 11; * $p < 0.05$, ** $p < 0.01$, *** $p < 0.001$, **** $p < 0.0001$; error bars represent s.d.)

Biology Open • Supplementary information

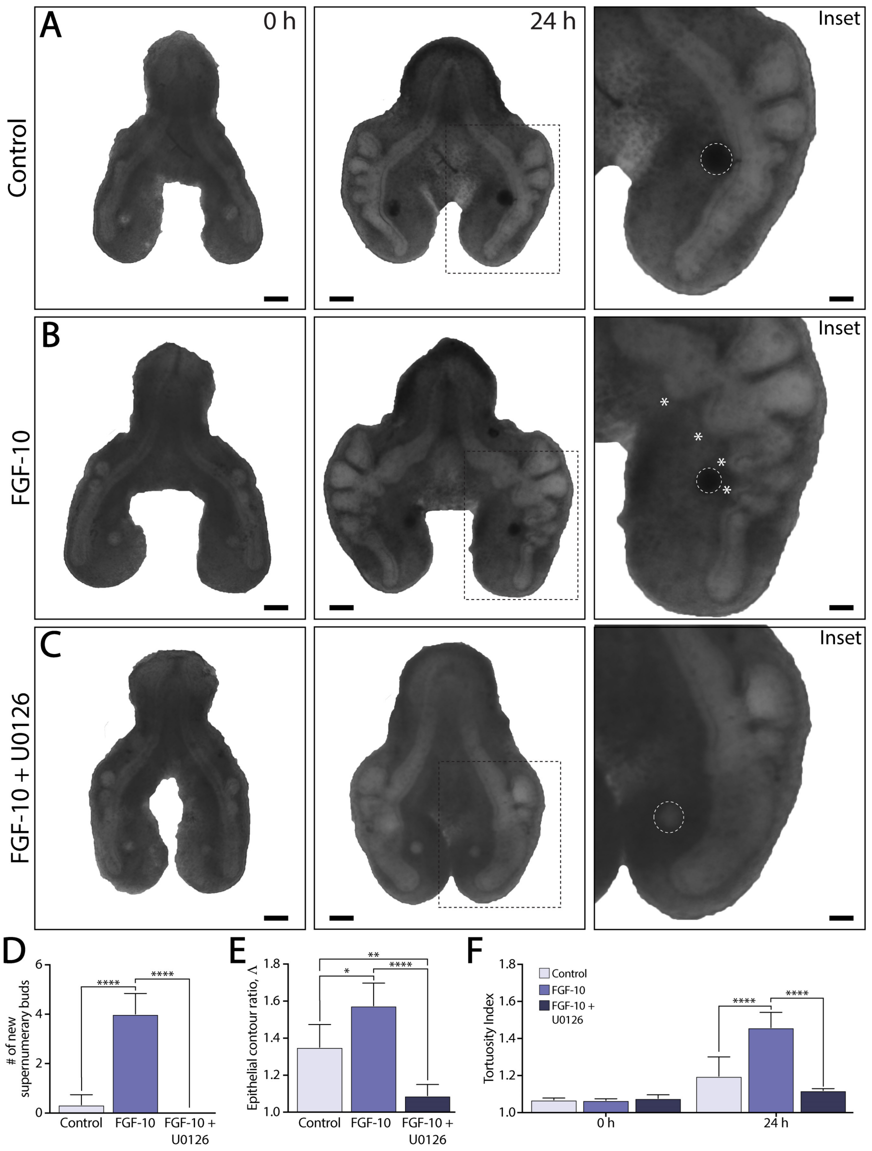

Fig. S5. MAPK inhibition prevents supernumerary budding morphogenesis. (A-C) Bright-field images of representative lung explants cultured ex vivo with either (A) PBS- or (B-C) FGF- 10-loaded beads. Some explants (C) were treated with MAP kinase inhibitor, U0126 (C). White dashed lines indicate the locations of growth-factor-loaded beads. White asterisks indicate super-numerary buds. Scale bars, 200 µm. (Inset scale bars, 100 µm.) (D-F) Quantification of (D) the number of supernumerary buds, (E) epithelial contour ratio, and (F) tortuosity index. A one-way (D-E) or two-way ANOVA (F), followed by a Tukey post-hoc test, was used to make statistical com-parisons. (PBS: n = 6, FGF-10: n = 6; FGF-10 + U0126: n = 6; * $p < 0.05$, ** $p < 0.01$, *** $p < 0.001$, **** $p < 0.0001$; error bars represent s.d.)

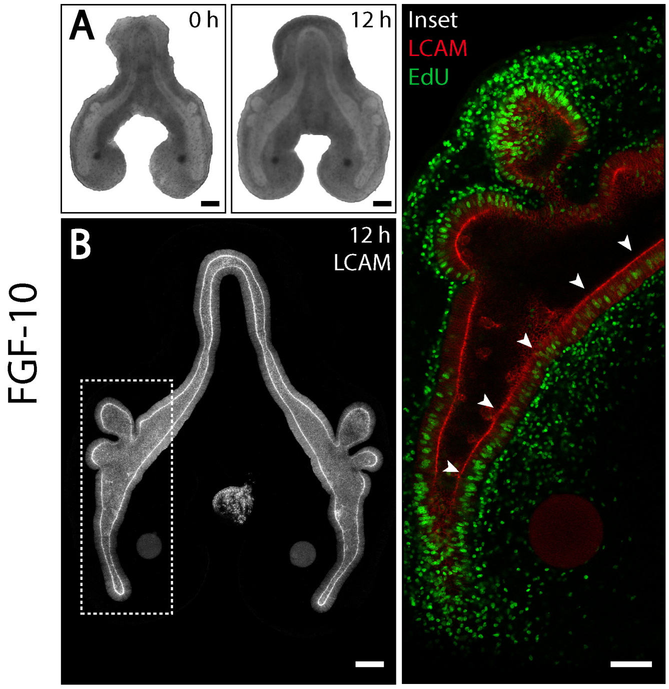

Fig. S6. No repeating pattern of localized proliferation is present before the emergence of supernumerary branches. (A) Bright-field images of representative lungs cultured ex vivo with FGF-10-loaded beads at 0 and 12 hr of culture. Scale bar, 200 µm. (B) Confocal image of LCAM immunofluorescence within representative lung explant fixed after 12 hr of culture. Inset panel (white dashed box) shows EdU incorporation and LCAM staining at higher magnification. No repeating pattern of localized proliferation could be observed along the ventral airway epithelium (white arrowheads) prior to the formation of supernumerary branches. Scale bar, 100 µm. (Inset scale bar, 50 µm)

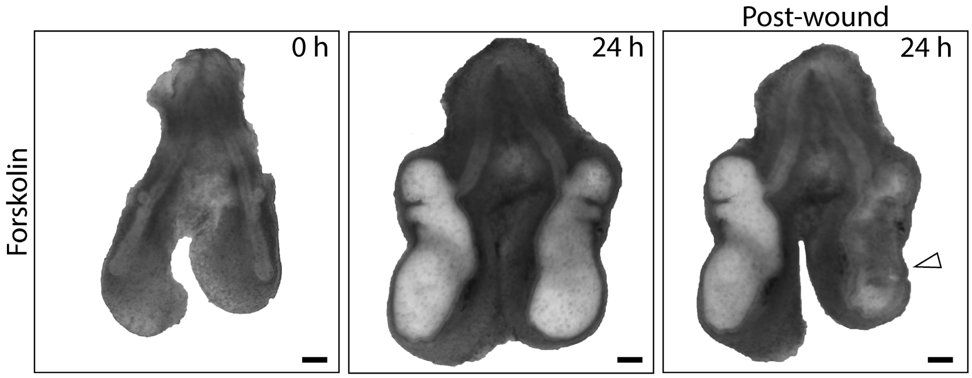

Fig. S7. Mechanically puncturing the airway in forskolin-treated lungs causes the primary bronchus to collapse. Representative images of an embryonic lung explant cultured in the presence of 10 µM forskolin at 0 and 24 hr. Following culture, a microneedle was used to puncture the airway in one lobe of the lung (white arrowhead), causing the primary bronchus to completely collapse. Scale bars, 200 µm.

Biology Open • Supplementary information

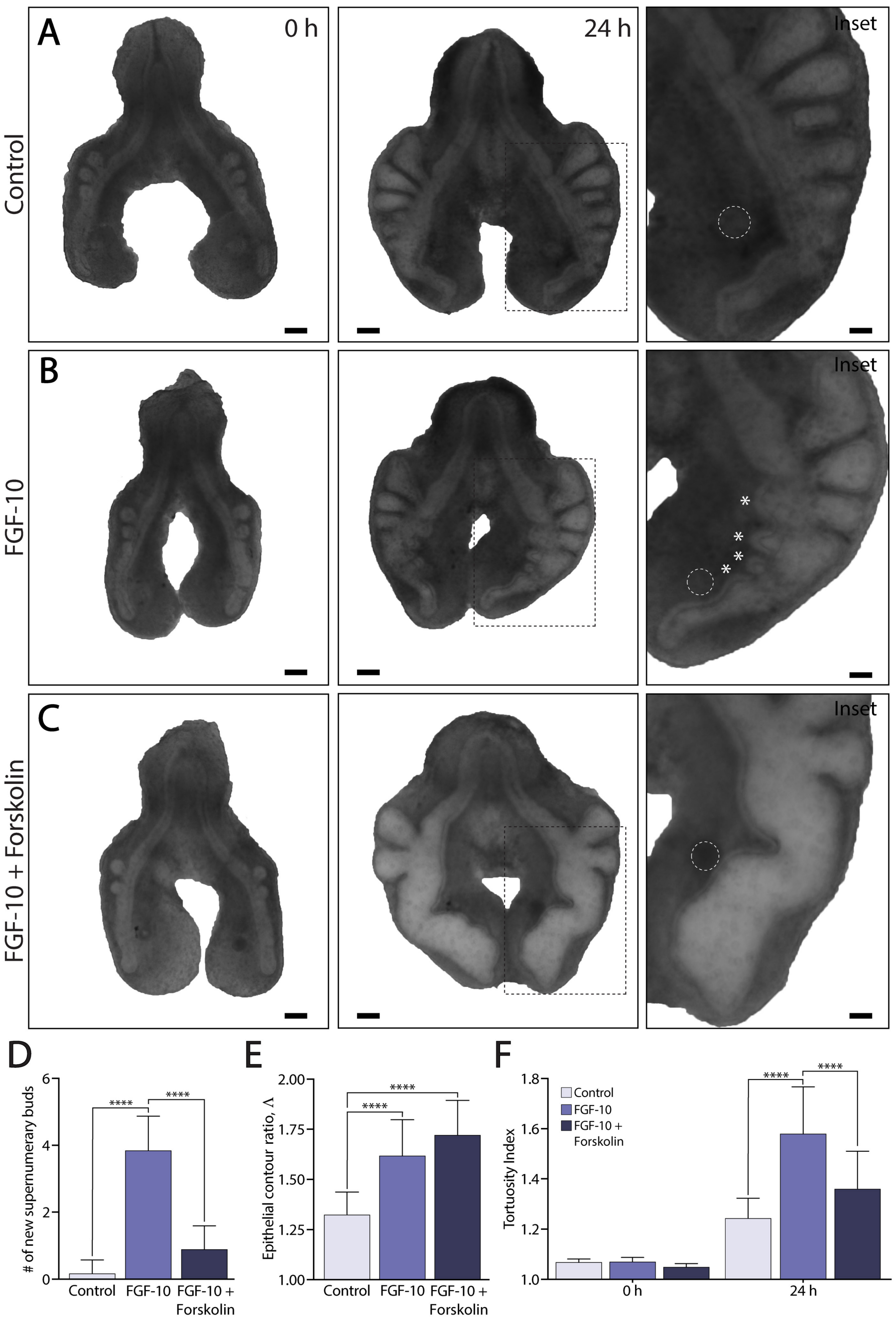

Fig. S8. Increased epithelial fluid secretion inhibits supernumerary budding morphogenesis. (A-C) Bright-field images of representative lung explants cultured ex vivo with either (A) PBS- or (B-C) FGF-10-loaded beads. Some explants (C) were treated with forskolin, an agonist of epithelial fluid secretion. White dashed lines indicate the locations of growth-factor-loaded beads. White asterisks indicate supernumerary buds. Scale bars, 200 µm. (Inset scale bars, 100 µm.)

Biology Open • Supplementary information

(D-F) Quantification of (D) the number of new supernumerary buds, (E) epithelial contour ratio, and (F) tortuosity index. A one-way (D-E) or two-way ANOVA (F), followed by a Tukey post-hoc test, was used to make statistical comparisons. (PBS: n = 12, FGF-10: n = 15; FGF-10 + forskolin: n = 14; * $p < 0.05$, ** $p < 0.01$, *** $p < 0.001$, **** $p < 0.0001$; error bars represent s.d.)

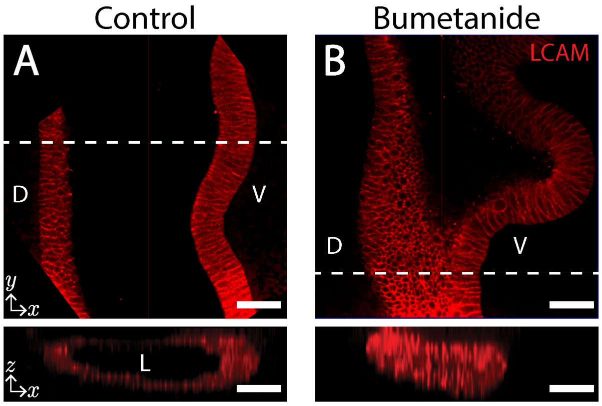

Fig. S9. Inhibiting epithelial fluid secretion produces a collapsed airway lumen. (A-B) Representative confocal images of LCAM immunofluorescence in embryonic lung explants treated with bumetanide. Transverse cross-sections (indicated by the dashed white line) show the airway lumen (L) in either (A) control or (B) bumetanide-treated explants. Note the collapsed lumen in (B). Scale bars, 25 µm. (V, ventral; D, dorsal)

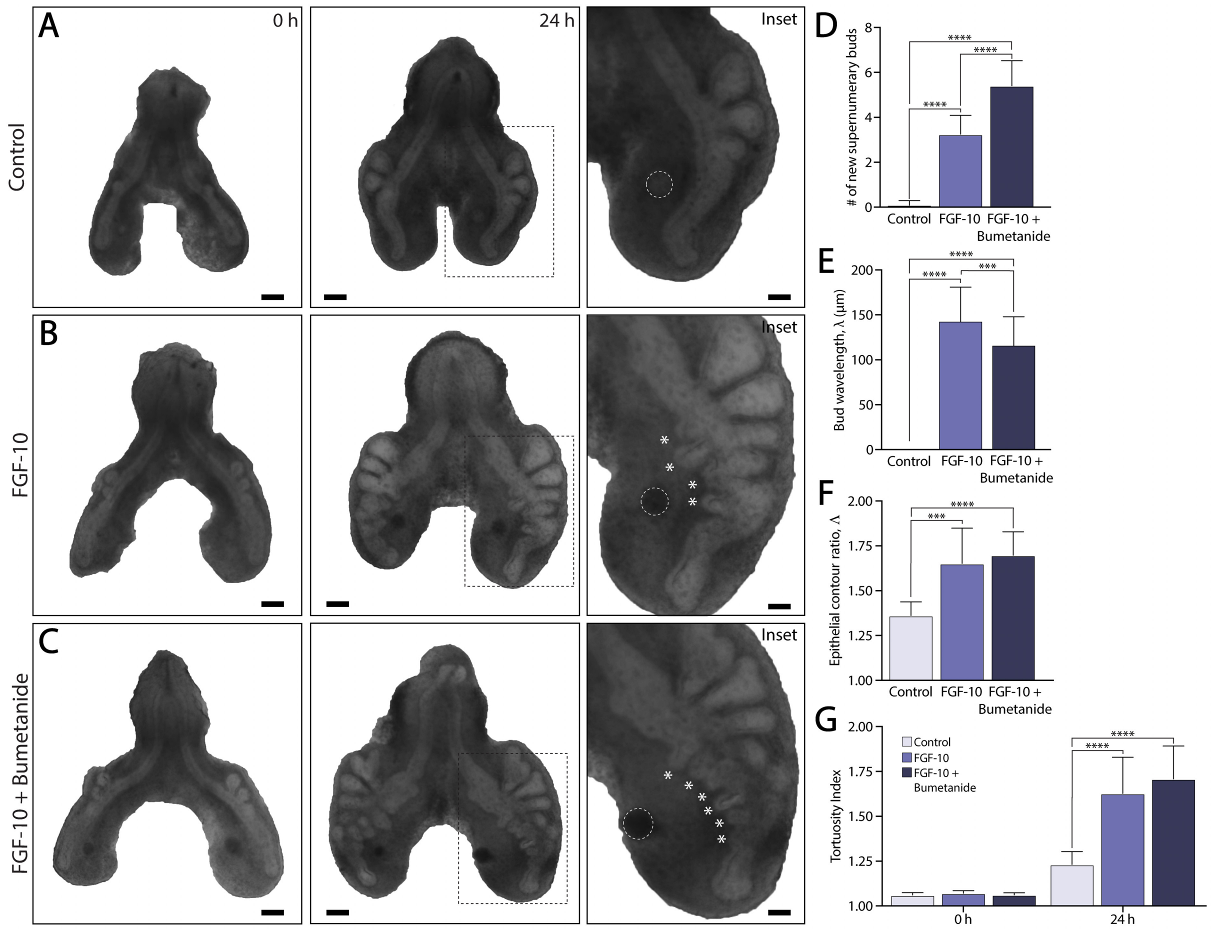

Fig. S10. Decreased epithelial fluid secretion promotes the formation of supernumerary buds. (A-C) Bright-field images of representative lung explants cultured ex vivo with either (A) PBS- or (B-C) FGF-10-loaded beads. Some explants (C) were treated with bumetanide, an inhibitor of epithelial fluid secretion. White dashed lines indicate the locations of the growth-factor-loaded beads. White asterisks indicate supernumerary buds. Scale bars, 200 µm. (Inset scale bars, 100 µm.) (D-G) Quantification of (D) the number of supernumerary buds, (E) bud wavelength, (F) epithelial contour ratio, and (G) tortuosity index. A one-way (D-F) or two-way ANOVA (G), followed by a Tukey post-hoc test, was used to determine differences among groups. (PBS: n = 11, FGF-10: n = 12; FGF-10 + bumetanide: n = 11; * $p < 0.05$, ** $p < 0.01$, *** $p < 0.001$, **** $p < 0.0001$; error bars represent s.d.)

Biology Open • Supplementary information

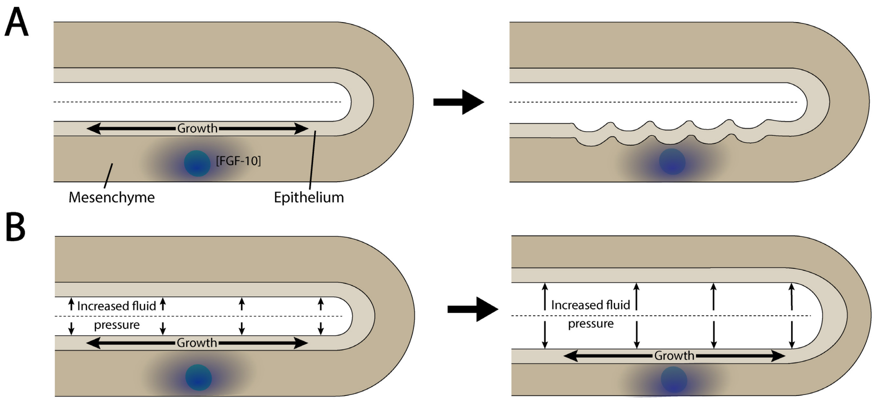

Fig. S11. Schematic of FGF-10-induced epithelial buckling. (A) A focal source of FGF-10 stimulates constrained growth within the ventral epithelium, which causes it to buckle and form multiple new supernumerary buds. (B) Increased luminal fluid pressure, however, suppresses epithelial buckling and FGF-10-induced budding morphogenesis.

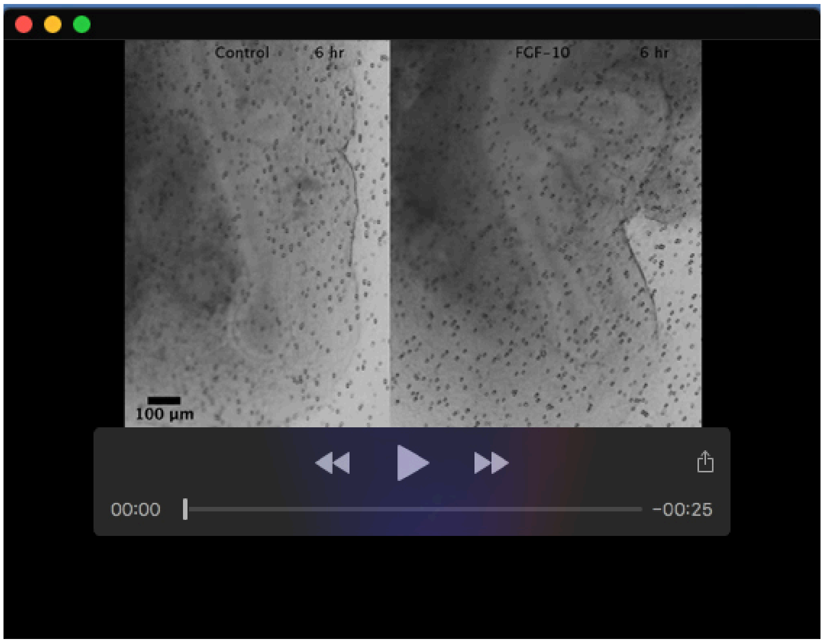

Movie 1. Time-lapse movie of embryonic lung explants cultured ex vivo with either PBS- (con-trol) or FGF-10-loaded beads. Bright-field images were captured every 30 min for 24 hr of culture. Scale bar, 100 µm.

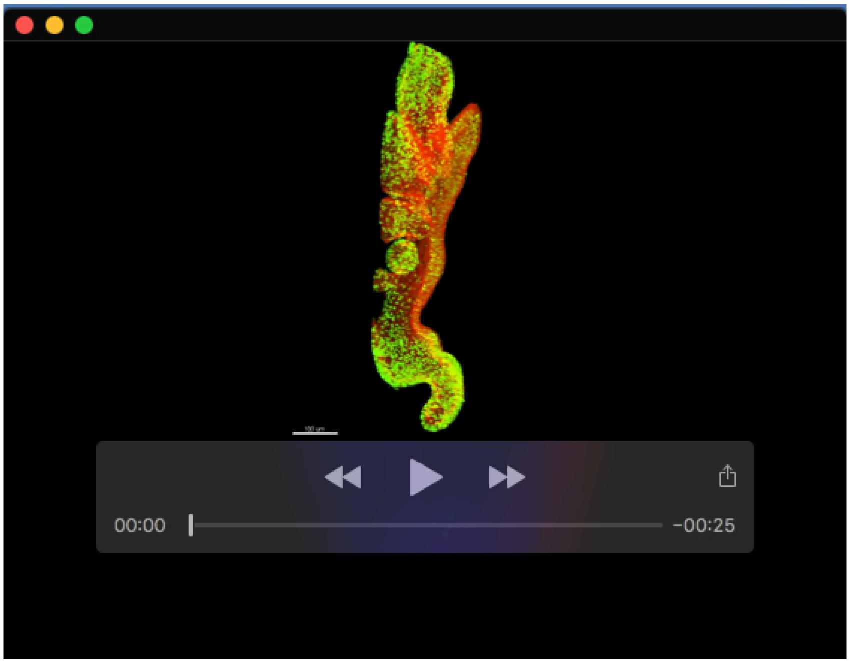

Movie 2. 3D reconstruction of EdU incorporation (green) and LCAM immunofluorescence (red) in a representative lung explant cultured with a PBS-soaked bead. In each confocal image, the LCAM channel was used to segment the population of EdU-positive nuclei in the airway epithelium. Scale bar, 100 µm.

Biology Open • Supplementary information

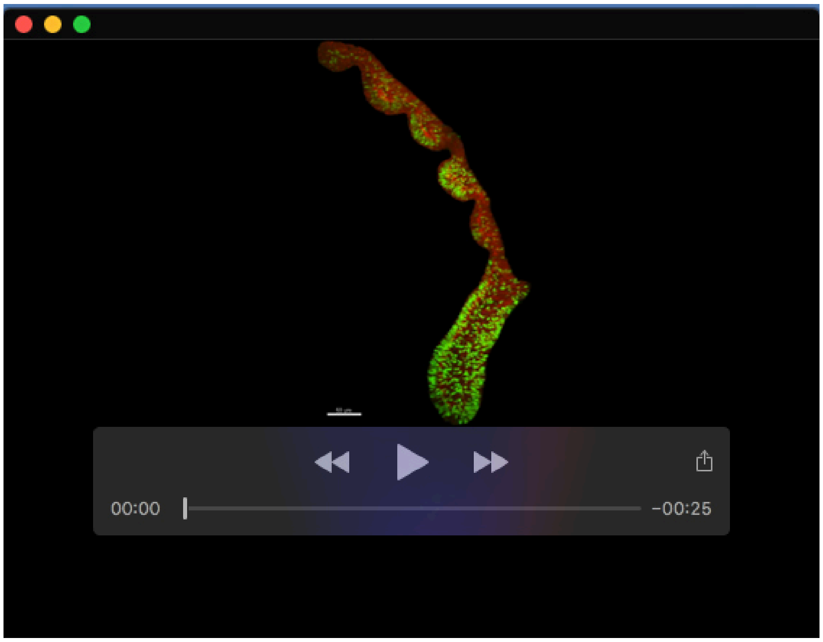

Movie 3. 3D reconstruction of EdU incorporation (green) and LCAM immunofluorescence (red) in a representative lung explant cultured with an FGF-10-loaded bead. In each confocal image, the LCAM channel was used to segment the population of EdU-positive nuclei in the airway epithelium. Scale bar, 100 µm.

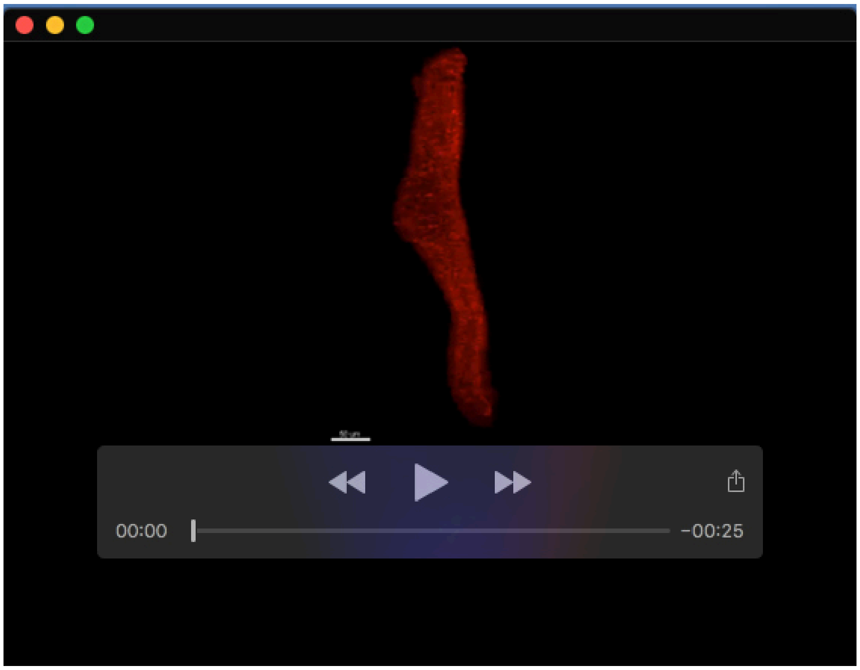

Movie 4. 3D reconstruction of EdU incorporation (green) and LCAM immunofluorescence (red) in a representative lung explant cultured with an FGF-10-loaded bead and treated with aphidicolin to inhibit cell proliferation. Note that no EdU-positive nuclei are present within the airway epithe-lium. Scale bar, 100 µm.

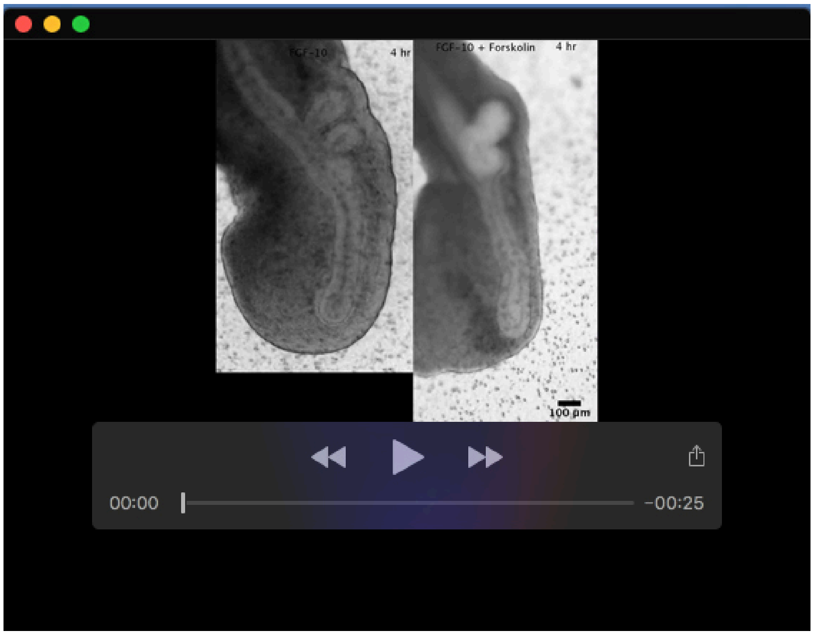

Movie 5. Time-lapse movie of embryonic lung explants cultured ex vivo with an FGF-10-loaded bead in either the presence or absence of forskolin, an agonist of epithelial fluid secretion. Bright-field images were captured every 30 min for 24 hr of culture. Scale bar, 100 µm.

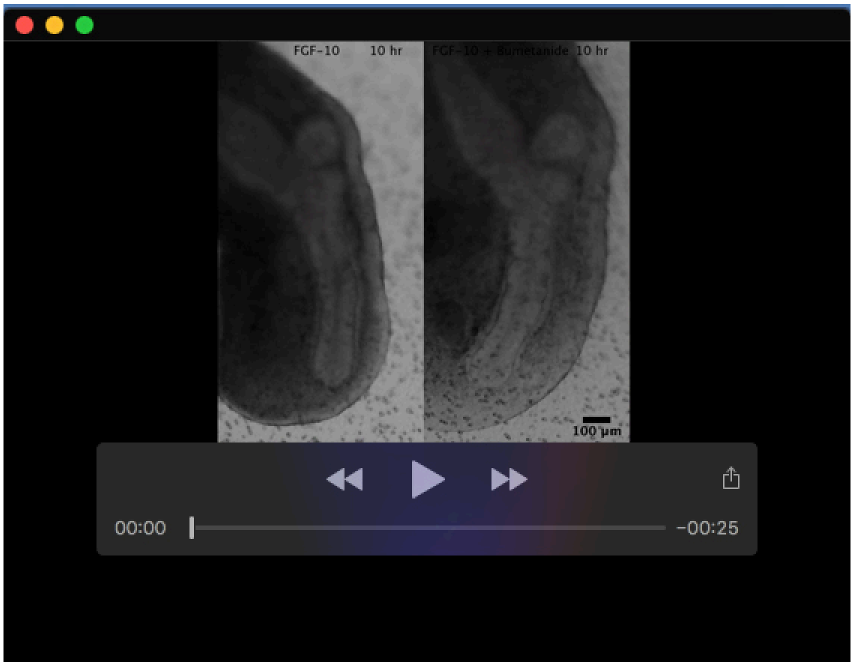

Movie 6. Time-lapse movie of embryonic lung explants cultured ex vivo with an FGF-10-loaded bead in either the presence or absence of bumetanide, an inhibitor of epithelial fluid secretion. Bright-field images were captured every 30 min for 24 hr of culture. Scale bar, 100 µm.

Biology Open • Supplementary information
